# 局限期小细胞肺癌预防性全脑照射：争议与研究进展

**DOI:** 10.3779/j.issn.1009-3419.2013.07.08

**Published:** 2013-07-20

**Authors:** 晓 胡, 明 陈

**Affiliations:** 310022 杭州，浙江省肿瘤医院放疗科 Department of Radiation Oncology, Zhejiang Cancer Hospital, Hangzhou 310022, China

小细胞肺癌（small cell lung cancer, SCLC）恶性度高，易远处转移，脑是最常见转移部位。由于常规化疗药难以有效进入脑组织，脑也就成了潜在转移灶的“庇护所”。因此，预防性脑照射（prophylactic cranial irradiation, PCI）并非起“预防”转移发生的作用，而是消灭尚不能被发现的潜在转移灶。

随着综合治疗的进步，SCLC患者生存率提高，存活患者增多，发生脑转移的几率也相应增高。Arrigada等^[1]^指出经治疗后疾病达到完全缓解（complete response, CR）的SCLC患者，2年内脑转移率为67%。发生脑转移的患者预后较差，中位生存时间仅约4个月-5个月^[2]^，且受损的神经功能难以恢复，生活质量受负面影响。另一方面，消灭亚临床转移灶所需放射剂量远低于消灭肉眼可见肿瘤所需剂量，而后者则可能已超出正常脑组织的耐受范围。

因此，对疾病得到控制的SCLC患者有必要积极行PCI，不应等到脑转移发生后再被动姑息放疗。但目前具体实施过程中在以下几方面尚存争议。

## 1 哪些患者需要接受PCI

早在20世纪70年代初，人们就已认识到了PCI对控制脑转移的重要性。但在一些早期研究中，由于入组患者临床异质性较大而未能得出PCI可以提高生存的结论。1983年，Rosen等^[3]^提出治疗后达到CR的患者才可能从PCI中获益。Auperin等^[4]^的荟萃分析纳入987例患者，显示对治疗后CR的患者行PCI较对照组3年生存率提高5.4%（20.7% *vs* 15.3%, *P*=0.01），并且降低16%（±6%）的死亡风险，且能降低累计脑转移发生率25.3%（33.3% *vs* 58.6%, *P* < 0.001）。对该研究随访更新后的结果仍得出PCI有利于提高生存率的结论：3年生存率PCI组较对照组提高6%（21% *vs* 15%, *P*=0.01）。而Meert等^[5]^随后的荟萃分析纳入了12项随机对照研究，共1, 547例患者，与Auperin等^[4]^的研究不同，所纳入的12项研究中仅5项针对治疗后CR的患者，结果表明接受PCI的患者发生脑转移的危险系数为0.48，但仅CR的患者有生存获益[危险比（hazard ratio, HR）=0.82，95%CI: 0.71-0.96]。

尽管这些研究发现治疗后CR的患者行PCI才有生存获益，但其纳入的研究有局限性，即疗效评价均采用胸部X光检查。因此这两项荟萃分析中部分CR患者实际疗效可能并非如此。而Slotman等^[6]^的研究表明，对广泛期SCLC治疗后有效的患者行PCI也有生存获益。

基于以上分析，人们推测在预后更好的局限期SCLC患者中，不仅对治疗后CR的患者，而且很可能对治疗后疗效评价部分缓解（partial response, PR）的患者行PCI也有生存获益。美国癌症与白血病研究组B（Cancer and Leukemia Group B, CALGB）正在进行的一项对比3种不同放疗剂量分割方式治疗局限期SCLC的Ⅲ期临床研究（CALGB30610/NCT00632853）中，将PCI的适应症定为对治疗有效的患者。

美国中北癌症治疗组^[7]^回顾性分析了化疗±胸部放疗后疗效稳定以上的739例SCLC患者，其中459例接受PCI，其生存时间明显长于未接受者（HR=0.61, 95%CI: 0.52-0.72, *P* < 0.000, 1）。且对年龄、PS状态、性别、分期、CR以及转移灶数目做调整后仍得出PCI有益的结论。

临床实践中，常有患者因肺内结节而手术，术后病理显示为SCLC，AJCC/UICC分期为Ⅰ期-Ⅱ期且病理完全切除，这些患者可能长期存活，而出于对PCI所致神经毒性的担心，部分医生对这部分患者是否应接受PCI提出疑问。

两项回顾性研究^[8, 9]^报道了手术切除的早期SCLC患者的随访结果，病理分期Ⅰ期的患者脑转移作为首发失败的发生率为7%-11%，Ⅱ期患者为25%-38%，显示早期SCLC的脑转移发生率仍不可小觑。Bischof等^[10]^回顾了39例手术完全切除的Ia期-IIb期SCLC患者，接受PCI的21例，随访结束时未发现脑转移，而未接受PCI的18例，其中4例（22.2%）在随访至8个月-27个月时发生脑转移; 无脑转移生存（*P*=0.01）及总生存（*P*=0.01）在接受PCI的患者均明显优于未接受者，作者因此建议对手术切除的Ia期-IIb期患者也应该行PCI。Patel等^[11]^回顾分析了7, 995例局限期SCLC患者，其中670例接受PCI，2年、5年、10年总生存率在进行和未行PCI的患者分别为42%、19%、9%和23%、11%、6%（*P* < 0.001）。亚组分析还发现按AJCC（第6版）分期为早期（Ⅰ期-Ⅱ期）的患者共2, 481例（31%），接受PCI者（188例）总生存率明显高于未接受PCI者（*P*=0.000, 8）。此外研究还发现不同性别、年龄段和种族的患者接受PCI均较未接受者有明显临床获益。但该研究的不足之处在于早期患者并非都接受了手术治疗。

Gong等^[12]^回顾分析了手术治疗的Ⅰ期-Ⅲ期SCLC患者126例，术后病理分期Ⅰ期、Ⅱ期、Ⅲ期脑转移发生率分别为6.25%（2/32）、28.2%（11/39）和29.1%（16/55）（*P*=0.026）。脑转移的发生与年龄、性别、病理类型、诱导化疗、辅助放化疗均无关。研究者因此认为完全切除的病理Ⅰ期SCLC发生脑转移风险较小，可不予PCI，但Ⅱ期、Ⅲ期患者仍应予PCI。

由此可见，对早期SCLC完全手术切除后的患者是否需要接受PCI尚存争议，值得进一步研究。

## 2 何时开始进行PCI

局限期SCLC开始PCI的最佳时间同样存在争议。Manapov等^[13]^回顾了105例局限期SCLC患者中的40例在放化疗后达到CR，但在准备行PCI之前复查MRI，发现13例（32.5%，95%CI: 18%-47%）发生脑转移，其中11例无症状。这13例患者中位生存时间为14个月（95%CI: 10.6-17.3），1年生存率为58.7%±14.2%，而余下未发生脑转移的27例患者中位生存时间为26个月（95%CI: 20.9-31.0），1年生存率92%±5.4%（*P*=0.000, 1）。发生和未发生脑转移的患者其预后差异明显。值得注意的是，该研究中PCI施行的时间为确诊疾病后4个月-10个月。研究表明，SCLC微小脑转移灶的倍增时间为3.5 d-16 d，远快于肉眼可见转移病灶的79 d。因此有观点认为尽量缩短PCI与诱导治疗的间隔有助于减少脑转移发生。

Schild等^[14]^的研究中，采用PCI早期介入，于6程化疗中的第3程评价疗效，只要有效便给予25 Gy/10次照射的方法，结果显示61例接受PCI的患者只有1例（1.6%）发生脑转移，但75%患者发生4度血液学毒性。由于颅骨骨髓占人体骨髓的12%，PCI与化疗相叠加必然导致严重血液学毒性。且多数研究^[15, 16]^报道PCI后神经系统毒性与同期化疗有关。因此，PCI不应与化疗同时进行，建议可以在化疗及胸部放疗结束后4周进行^[17]^。前述CALGB30610/NCT00632853研究也将PCI的时间定为胸部放疗及化疗结束后3周-6周开始。

## 3 PCI的剂量/分割

局限期SCLC行PCI的最佳放疗剂量尚在探索中。Auperin等^[4]^的荟萃分析间接比较了4种不同的放疗剂量（8 Gy、24 Gy-25 Gy、30 Gy及36 Gy-40 Gy），发现剂量越高，脑转移的风险越低（*P*=0.01）。但各种不同放疗剂量间对生存的影响差异无统计学意义。随后的一项Ⅲ期研究中^[18]^，720例治疗后CR的局限期SCLC患者随机分为接受25 Gy/10次、36 Gy/18次或36 Gy/24次（1.5 Gy，2次/d）放疗。较高剂量组2年脑转移累积发生率较标准剂量组低，分别为23%（95%CI: 18%-29%）和29%（95%: 24%-35%），但差异无统计学意义（HR=0.8, 95%CI: 0.57-1.11, *P*=0.18）。RTOG 0212^[19]^研究中对这3种剂量分割方式所造成的慢性神经毒性及对生活质量的影响做了分析，发现在PCI后12个月时36 Gy剂量组发生慢性神经毒性较25 Gy剂量组明显增加（*P*=0.02）。

前述美国中北癌症治疗组^[7]^的研究还发现对局限期SCLC患者而言采用25 Gy/10次放疗较30 Gy/15次可有明显生存获益（HR=0.67, *P*=0.018），但作为回顾性研究，该结果不能作为定论。因此，目前局限期SCLC行PCI标准剂量仍为25 Gy/10次。虽然NCCN治疗指南中也推荐30 Gy/15次，但从方便及经济的角度来说，前者是较好选择。

## 4 PCI的潜在神经系统毒副作用

虽然目前的研究表明PCI有明确生存获益，但放疗中可能出现的急性毒性及在长期生存的患者中可能引起的放射后期反应，不仅是放疗科医生，也普遍是肿瘤内、外科医生们关注的问题。

由PCI所致的常见急性反应有脱发、皮肤红斑、乏力、头痛、恶心、呕吐及情绪变化，由于放疗时常规予以脱水等对症处理，严重的脑水肿、颅内高压较少见，其它急性反应常也轻微，经对症处理常可以取得良好缓解。文献中所报道的后期放射反应有记忆力下降，智力障碍甚至痴呆以及共济失调等。脑CT/MRI检查可见脑萎缩、脑室扩张、脑室周围及脑皮质下白质异常等，但影像学改变不意味着一定会出现神经认知功能障碍。需指出的是，很多有关PCI神经毒性的报道为小样本量的回顾性研究，没有记录治疗前的基线神经认知功能状态。而影响患者神经认知功能的原因除PCI之外还有如化疗的使用、年龄、脑血管危险因素、糖尿病、长期吸烟、对疾病本身的焦虑及一些副瘤综合症等。因此这些研究中神经毒性与PCI的潜在相关性需谨慎看待^[20-23]^。

Arriagada等^[1]^的研究是有关PCI的大型前瞻性随机对照研究中最早关注放疗相关神经毒性的。研究入组294例治疗后疗效评价达CR的局限期SCLC患者，随机分为治疗组（24 Gy/8次）或观察组。两组均应用神经心理学测试及脑CT检查在治疗前后对患者进行评价，结果发现两组间神经系统功能受损的发生几率、CT显示脑萎缩和脑室扩张的发生率差异均无统计学意义。

Gregor等^[24]^的前瞻性随机研究也关注了患者认知功能及生活质量问题。研究入组314例患者，治疗前及治疗后每半年进行测试。认知功能损害以年龄配对对照的差异来定义。观察发现多达40%的患者在治疗前即存在一定程度的神经认知功能障碍及生活质量受损。而在治疗后的随访中两组差异无统计学意义，未显示出PCI有明显副作用。

Grosshans等^[25]^的前瞻性研究对局限期SCLC患者行PCI前后神经认知功能检查，发现约47%的患者在接受PCI前已经有认知功能的受损。在接受PCI后，单因素分析发现执行功能及语言功能会发生一过性下降，之后恢复; 多因素分析则未发现PCI前后的差别。

Le Pechoux等^[26]^的研究的同时也评价了25 Gy和36 Gy两种不同剂量照射对患者神经系统及生活质量的影响。患者在治疗前、治疗后半年、1年及以后每年1次填写生活质量问卷调查表（QLQ-C30/BN20）^[27, 28]^及接受MRI或CT检查; 同时根据EORTC-RTOG正常组织后期反应等级^[29, 30]^对患者功能与认知状态进行分级。结果显示两个不同剂量组间各指标差异无统计学意义。但研究发现年龄大于60岁-65岁是发生神经认知功能障碍的危险因素。

RTOG 0212研究^[18]^中则发现60岁以上的患者中有83%在接受PCI后1年会发生神经系统毒性反应，而60岁以下的患者发生率为56%（*P*=0.009）。

有研究认为大脑海马部位的齿状回是神经元干细胞聚集的位置，而全脑放疗后这个位置的损伤是造成神经认知功能障碍的关键。Gondi等^[31]^采用断层放疗技术或IMRT技术以期尽量保护海马而不影响大脑其它部位剂量分布。结果显示给予30 Gy/10次放疗时可以降低该部位中位剂量5.5 Gy-7.8 Gy，最多可降低12.8 Gy-15.3 Gy。该作者还回顾了371例患者，1, 133个转移病灶中海马区域周围5 mm范围发生转移的几率为3%，占所有患者数的8.6%，但海马区本身未观察到转移^[32]^。因此这种技术手段被认为是安全的，但仍需要进一步于临床研究中验证。

综上所述，目前已有的研究证据支持对放化疗后达CR及PR的局限期SCLC患者行PCI。放疗可考虑于胸部放化疗结束后4周进行，不宜推迟太久。实施PCI前需再次评估包括脑MRI在内的全身肿瘤状况以排除已有脑或其他器官转移及无明显治疗效果的患者。推荐放疗剂量为25 Gy/10次或30 Gy/15次。对已经有明显神经认知功能障碍及一般状态差（PS评分为3分-4分）的患者不推荐行PCI。由于目前鲜有针对高龄患者的研究，因此对这些患者在决定行PCI之前须仔细了解患者病史、内科合并症情况、评估神经认知功能状态等以及结合患者本人意愿，尽量给予个体化的治疗。
